# Inhibition of LPS-Induced Activation of Coagulation by p38 MAPK Inhibitor

**DOI:** 10.5402/2012/762614

**Published:** 2012-03-05

**Authors:** Lutz Koch, Stefan Hofer, Markus A. Weigand, David Frommhold, Johannes Poeschl, Peter Ruef

**Affiliations:** ^1^Division of Neonatology, Department of Paediatrics, Medical School, University of Heidelberg, Im Neuenheimer Feld 430, 69120 Heidelberg, Germany; ^2^Department of Anesthesiology, University of Heidelberg, Im Neuenheimer Feld 110, 69120 Heidelberg, Germany; ^3^Department of Anesthesiology, University of Giessen, Rudolf-Buchheim-Str. 7, 35392 Giessen, Germany

## Abstract

During Gram-negative sepsis, lipopolysaccharide (LPS) activates toll-like receptor (TLR) 4 and induces complex responses of immune system and coagulation. However, the underlying LPS signalling mechanism on coagulation activation remains complex. To determine the role of the intracellular signalling factors p38 mitogen-activated protein kinase (MAPK), nuclear factor-kappa B (NF-**κ**B), and c-Jun N-terminal kinase (JNK) in the procoagulant response to LPS, coagulation process of human whole blood exposed to specific inhibitors was measured by thrombelastography. Samples were stimulated with LPS (100 **μ**g/mL) after preincubation with BAY117082 (specific NF-**κ**B inhibitor), SP600125 (specific JNK inhibitor), SB203580 (specific p38 MAPK inhibitor), or vehicle. SB203580 strongly inhibited LPS-induced coagulation activation, whereas BAY117082 and SP600125 showed no significant effect. Activation of p38 MAPK, NF-**κ**B, and JNK and respective inhibitory effects were confirmed by Multi-Target Sandwich ELISA. In conclusion, activation of p38 MAPK is crucial for early LPS-induced activation of coagulation.

## 1. Introduction

Activation of the coagulation system is an important part of the systemic inflammatory response during infection. Lipopolysaccharide (LPS), a complex outer membrane glycolipid of Gram-negative bacteria, triggers an array of cellular activations through a variety of mechanisms involving diverse intracellular signalling [1–3]. In patients with Gram-negative sepsis bacterial LPS induces the expression of tissue factor (TF) on monocytes and endothelial cells, leading to disseminated intravascular coagulation [4, 5]. However, mechanisms involved in LPS-induced coagulation activation remain complex. Binding of LPS to toll-like receptor (TLR) activates several intracellular signalling cascades that include nuclear factor-kappa B (NF-*κ*B), c-Jun N-terminal kinase (JNK), and p38 mitogen-activated protein kinase (MAPK) [6, 7]. The roles of NF-*κ*B, JNK, and p38 MAPK in activation of coagulation in vivo are not completely known. Therefore the present study was designed to investigate the effects of BAY117082, SP600125, and SB203580, specific and potent inhibitors of NF-*κ*B, JNK, and p38 MAPK, in the procoagulant response to LPS in healthy subjects measured by thrombelastography (TEG).

## 2. Materials and Methods

### 2.1. Blood Sampling and Incubation of Whole Blood Samples

After receiving written informed consent, venous blood from eleven healthy volunteers was collected in one-tenth volume of citrate (3.8%, Becton Dickinson, Heidelberg, Germany) and immediately used for the experiments. Blood samples were incubated with 1, 10, or 100 *μ*M BAY117082, SP600125, SB203580, or vehicle for 1 h. Thereafter, 100 *μ*g/mL Escherichia coli LPS (Serotype 0111 : B4, Sigma-Aldrich, Germany) was added for further 15 min in ELISA experiments or further 4 h in TEG experiments. All incubations were performed at 37°C.

### 2.2. Determination of Clotting Time and Clot Firmness

Whole blood clotting time (CT) and maximal clot firmness (MCF) were determined as described [8]. In short, citrated whole blood samples containing LPS and a specific inhibitor, as stated in the respective experiments, were recalcified with calcium chloride and subjected to rotational thrombelastography (Roteg 5, Pentapharm, Munich, Germany), a modification of the original thrombelastography method [9]. 

### 2.3. Inhibition of NF-*κ*B p65, p38 MAPK, and JNK Signalling Pathways

Following incubation period reaction was stopped by Lyse/Fix Buffer (BD Phosflow, BD, San Jode, USA). After a wash with phosphate-buffered saline, protein lysates were prepared using the lysis buffer included in the PathScan Inflammation Multi-Target Sandwich ELISA Kit (Cell Signaling, Danvers, USA). The lysates were subjected to ELISA analysis for the phosphorylated forms of NF-*κ*B p65, p38 MAPK, and JNK in duplicate using the ELISA Kit according to the manufacturer's instructions.

### 2.4. Statistical Analysis

All results are given as mean and standard deviation. The normality distribution was tested using Kolmogorov-Smirnov test showing that all variables were normally distributed. The results were evaluated using one-way analysis of variance (ANOVA). Statistical significance was set at *P* < 0.05. After testing homogeneity of variance by Levene test, the Tukey HSD post hoc test was selected for all analyses. All analyses were done using PASWStatistics 18.0 (SPSS Inc., Chicago, IL, USA).

## 3. Results

### 3.1. Phosphorylation of p38 MAPK, NF-*κ*B, and JNK after LPS Stimulation with or without Preincubation with Specific Inhibitors

**Figure 1:**
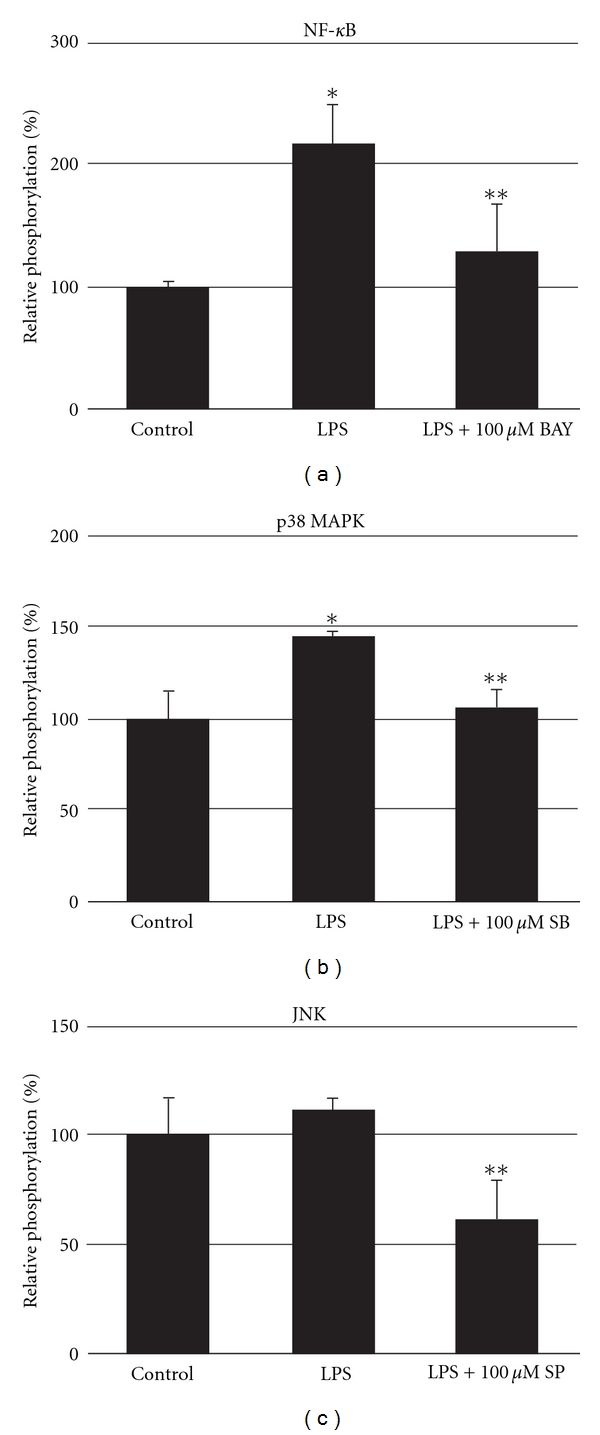
Phosphorylation of p38 MAPK, NF-*κ*B, and JNK after LPS stimulation with or without preincubation with specific inhibitors. Whole blood was stimulated with LPS (100 *μ*g/mL) for 15 min. After fixing and washing, protein lysates were prepared and subjected to ELISA analysis for phosphorylated forms of p38 MAPK, NF-*κ*B, and JNK. Means ± SD of optical densities are expressed as the levels of activation relative to controls (set to 100%). *P < 0.05 versus data for control; **P < 0.05 versus data for LPS. BAY = BAY117082, SB = SB203580, SP = SP600125.

Stimulation of blood with 100 *μ*g/mL LPS induced rapid phosphorylation of NF-*κ*B and p38 MAPK within 15 min. NF-*κ*B phosphorylation increased 2-fold (217 ± 33%) and p38 MAPK phosphorylation increased 1.5-fold (145 ± 4%) (Figures 1(a) and 1(b)). In contrast, JNK showed no alteration in phosphorylation after incubation with LPS (Figure 1(c)). Preincubation with the p38 MAPK inhibitor SB203580 and NF-*κ*B inhibitor BAY117082 completely blocked the LPS-activated phosphorylation of p38 MAPK and NF-*κ*B, respectively. The JNK inhibitor SP600125 diminished JNK phosphorylation almost by half (Figure 1). The inhibition effect after LPS stimulation of all three inhibitors showed similar effects: BAY117082 reduced NF-*κ*B phosphorylation from 217% to 129%   (−41%), SB203580 reduced p38 MAPK phosphorylation from 145% to 105% (−28%), and SP600125 reduced JNK phosphorylation from 110% to 61% (−44%), respectively.

### 3.2. Clotting Time (CT) in Whole Blood after LPS Stimulation with or without Preincubation with Specific Inhibitors

**Figure 2:**
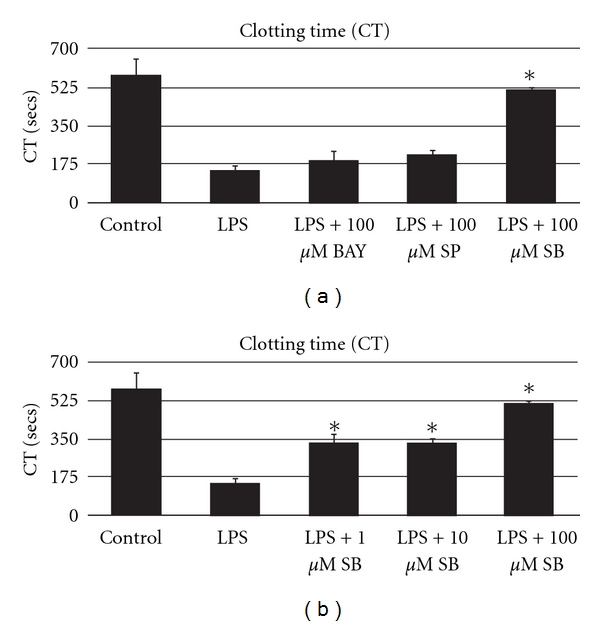
Clotting time (CT) in whole blood after LPS stimulation with or without preincubation with specific inhibitors. (a) Levels of CT of whole blood under control conditions, in the presence of LPS (100 *μ*g/mL) for 4 hours and after pretreatment with 100 *μ*M BAY117082 (BAY), 100 *μ*M SP600125 (SP), or SB203580 (SB) for 1 hour. Data are presented as mean ± SD. *P < 0.05 versus data for LPS. (b) Levels of CT of whole blood under control conditions, in the presence of LPS (100 *μ*g/mL) for 4 hours, and after pretreatment with 1 *μ*M, 10 *μ*M, or 100 *μ*M SB203580 (SB) for 1 hour. Data are presented as means ± SD. *P < 0.05 versus data for LPS.

During incubation of whole blood with 100 *μ*g/mL LPS over a period of 4 hours, we observed a reduction of CT from 579 ± 76 sec to 145 ± 25 sec (Figure 2). Preincubation with the p38 MAPK inhibitor SB203580 inhibited LPS-induced coagulation (Figure 2(b)). Preincubation with 1 *μ*M SB203580 increased CT from  145 ± 25 to 330 ± 114 sec, 10 *μ*M SB203580 to 329 ± 69 sec, and 100 *μ*M SB203580 to 512 ± 12 sec, respectively.

In contrast to the marked effects of SB203580, administration of BAY117082, a selective NF-*κ*B inhibitor, and SP600125, a selective JNK inhibitor, showed no significant effect on CT (Figure 2(a)). Preincubation with 100 *μ*M BAY117082 and 100 *μ*M SP600125 increased CT from 145 ± 25 sec to 191 ± 44 sec and from 145 ± 25 sec to 218 ± 26 sec, respectively.

## 4. Discussion

We recently demonstrated that inhibition of LPS-induced p38 MAPK activation in neonatal and adult blood was associated with a strong reduction in release of cytokines, whereas pharmacological inhibition of NF-*κ*B showed no effect [10]. In the present study we investigated the role of p38 MAPK, NF-*κ*B, and JNK for activation of hemostasis measured by TEG.

For the determination of coagulation in whole blood samples, we used TEG as reported previously [8]. Although fast and simple, TEG is able to analyse and dissect single steps of the dynamic process of blood coagulation differentially, beginning from activation of clotting factors through fibrin formation, platelet aggregation, and, finally, clot lysis [11]. The sensitivity of this method is high, as shown by concentration response curve for TF, because exogenously applied TF shortened clotting time at concentrations as low as 100 fM. This concentration is 60 times smaller than the TF concentration evoked by LPS in the present study [12]. We used LPS at a concentration of 100 *μ*g/mL to stimulate coagulation cascade. This concentration was chosen according to a concentration response curve which was constructed under identical experimental conditions [13]. The 50% effective concentration (EC50) of the LPS effect in that study was 18 *μ*g/mL, and this value corresponds well to the concentration range of other whole blood studies [14–16]. Therefore we decided to maximally stimulate coagulation using the fivefold concentration in the present study. The relevance of the chosen LPS concentration is underlined by the fact that the LPS content of erythrocytes from septic patients has been demonstrated by our group to be 77 ± 26 *μ*g/mL [17]. Furthermore, the procoagulant effects of LPS was demonstrated to be mediated by de novo synthesis of TF, since cycloheximide and active site-inhibited factor VIIa, respectively, completely inhibited the LPS-induced shortening of CT [13].

Stimulation of blood with LPS induced rapid phosphorylation of p38 MAPK and NF-*κ*B within 15 min, whereas JNK were not altered. Several authors have implicated NF-*κ*B and p38 MAPK to be critical mediators of the release of inflammatory cytokines and regulate the expression of a variety of genes involved in the acute-phase response such as TNF-*α*, IL-6, and other inducible enzymes [18, 19]. Preincubation with the p38 MAPK inhibitor SB203580 and NF-*κ*B inhibitor BAY117082 blocked the LPS-activated phosphorylation of p38 MAPK and NF-*κ*B to control values, respectively. The JNK inhibitor SP600125 diminished JNK phosphorylation almost by half. Concerning inhibition effect after LPS stimulation, SP600125 showed similar properties compared to SB203580 and BAY117082.

Incubation with LPS was associated with induction of coagulation cascade, as reflected by strong reduction of CT. Since tissue factor is essential for activation of the coagulation cascade [20, 21] and p38 MAPK inhibition reduces LPS-induced tissue factor, the anticoagulatory activity of SB203580 may be due to suppression of the LPS-induced tissue factor upregulation [22]. Furthermore, reduced proinflammatory response through p38 MAPK inhibition might have an additional inhibitory effect on activation of hemostasis [10, 23–25]. In contrast to the marked effects of SB203580 on CT, the inhibitor did not affect maximal clot firmness (MCF) (not depicted), which is dependent on fibrinogen polymerization, platelet number, and function, confirming the expected effect of SB203580 on tissue factor inhibition.

Although LPS strongly activates NF-*κ*B, administration of BAY117082, a selective NF-*κ*B inhibitor, showed no significant effect on CT. In line, we recently demonstrated that inhibition of NF-*κ*B had no significant effect on LPS-induced early cytokine expression in neonatal and adult whole blood [10].

Furthermore, LPS activation of cells of monocytic lineage rapidly activates the JNK pathway [26]. Many of the downstream targets of the JNK pathway are transcription factors including c-Jun, ATF-2, and Elk-1 which regulate transcription of proinflammatory mediators, like TF [27]. However, in the present study JNK pathway was not activated by LPS, and selective inhibition of JNK showed no effect on LPS-induced coagulation. These results are in line with recently flow cytometric analysis of human monocytes showing no significant changes in phosphorylation of JNK after LPS stimulation [28]. According to our knowledge all other studies, which showed LPS-mediated activation of JNK pathway, were done in (tumor) cell line models and therefore difficult to compare. We suggest that NF-*κ*B and JNK activation play a minor role in LPS-mediated early systemic inflammatory response and early activation of coagulation.

In conclusion we purport that p38 MAPK is crucially involved in early activation of coagulation during LPS stimulation. To confirm procoagulatory properties of p38 MAPK during inflammation further in vivo studies using specific inhibitors are necessary [29].
